# The earlier the initiation of gonadotropin in poor responders in luteal phase stimulation protocols, the better

**DOI:** 10.3389/fendo.2022.979934

**Published:** 2022-11-16

**Authors:** Jia Huang, Luxin Liu, Yue Wu, Benyu Miao, Yubin Li, Canquan Zhou, Yanwen Xu

**Affiliations:** ^1^ Reproductive Medicine Center, The First Affiliated Hospital, Sun Yat-Sen University, Guangzhou, Guangdong, China; ^2^ Guangdong Provincial Key Laboratory of Reproductive Medicine, The First Affiliated Hospital, Sun Yat-Sen University, Guangzhou, Guangdong, China

**Keywords:** luteal phase stimulation, timing of gonadotropin initiation, duration of ovarian stimulation, clinical outcome, poor responder

## Abstract

**Introduction:**

Luteal-phase ovarian stimulation has been proved to be feasible for producing competent oocytes/embryos and achieving live births, yet there is no standardized stimulation protocol for luteal-phase ovarian stimulation (LPS). The aim of this study was to explore the optimal timing of gonadotropin initiation in the LPS protocol for poor ovarian responders.

**Methods:**

This was a retrospective cohort study conducted in the reproductive medicine center of a tertiary hospital. A total of 327 poor responders fulfilling Bologna criteria underwent LPS with IVF/ICSI treatment. HMG and letrozole were administrated after ovulation. Patients were stratified into three groups according to the gonadotropin start day: early, early-mid, and mid-late luteal phase. A freeze-all strategy was performed for all cycles. The duration of ovarian stimulation, total gonadotropin dose, number of oocytes retrieved, implantation rate, clinical pregnancy rate, and live birth rate after frozen/thawed embryo transfer cycles were included for evaluation.

**Results:**

The group accepted ovarian stimulation in the earlier phase tended to have a shorter duration of ovarian stimulation [8 (7,10) in early luteal group, 9 (8,10.25) in early-mid luteal group, and 11 (10,12) in mid-late luteal group; *P <*0.001] and lower gonadotropin consumption [1993.35 ± 720.31, 2282.73 ± 703.38, and 2764.83 ± 722.26, respectively; *P <*0.001]. Logistic regression and multiple linear regression were used to assess the associations between the phase of gonadotropin initiation and duration of ovarian stimulation (or total gonadotropin dose) by adjusting for confounding factors. Compared with the early luteal group, longer ovarian stimulation(>9 days) was more likely to occur in the early-mid and mid-late luteal groups, with the adjusted odds ratios 0.584 (0.327-1.042) and 0.116 (0.049-0.271), respectively (*P*-trend<0.001). Delayed gonadotropin initiation showed an 113.200 IU increase (95%CI: 70.469, 155.930) per-day in the total gonadotropin dosage. Meanwhile, there were no significant differences in the mean number of oocytes, utilizable embryos, pregnancy outcomes among three groups.

**Conclusion:**

Although the timing of gonadotropin initiation is not associated with pregnancy outcomes, earlier initiation of gonadotropin therapy after ovulation was associated with a shorter duration of ovarian stimulation and lower gonadotropin consumption in poor responders in LPS.

## Introduction

Poor ovarian response after IVF treatments is a big challenge for both patients and clinicians (1). Poor responders tend to have low oocyte yields, high cancellation rates, as well as lower pregnancy and live birth rates (2). It is necessary to explore the optimal ovarian stimulation (COS) protocol in poor responders.

With the increasing knowledge of human folliculogenesis, luteal phase ovarian stimulation (LPS) as a new ovarian stimulation protocol has been introduced into IVF in the last decade. High progesterone level in luteal phase reduces GnRH pulsatile from the hypothalamus, thus inhibiting luteinizing hormone (LH) peak induced by increased estradiol levels, resulting in that GnRH analogs are not necessary for the stimulation in luteal phase. Therefore, the LPS protocol is associated with the promising advantages like convenience and low cost without the GnRH analogs for pituitary suppression (3). A series of trials have confirmed the feasibility of the LPS protocol. In 2014, Kuang et al. further proved the feasibility of LPS protocol with a study of 227 patients stimulating after spontaneous ovulation, for 68 live births and 44 ongoing pregnancies were achieved without any stimulation-related complications (4). Since then, increasing studies have showed that oocytes obtained after LPS are developmentally, genetically and reproductively competent (4–7). Another study showed that LPS did not cause an elevated rate of abnormalities at birth with the data to date (8). Although all fertilized oocytes or embryos must be cryopreserved for a later frozen-thawed embryo transfer cycle (FET) in such protocol, previous studies revealed that a frozen-thawed embryo transfer has similar treatment outcomes to a fresh embryo transfer (9).

According to previous studies, normal responders did not benefit significantly from the LPS. Normal responders had significantly longer days of stimulation and more gonadotropins with the LPS protocol compared to FPS protocol, but the mean number of oocytes retrieved, the number of M II oocytes, the cycle cancellation rates and clinical pregnancy rates were similar between the two protocols (10, 11). However, for poor responders, more oocytes,MII oocytes, fertilized oocytes, embryos, clinical pregnancy rate and implantation rate achieved during LPS than FPS (12). Furthermore, a recent randomized, open-label pilot trial in women with POR fulfilling Bologna criteria showed that the length of ovarian stimulation was similar between LPS (gonadotropin initial from the fourth day of the positive LH test) and FPS groups (8.35 ± 2.8 vs. 8.15 ± 4.1 days, *P* = 0.69) (13). A retrospective study including 69 women with severe DOR also showed no statistically significant difference in the total dose of gonadotropin administered (334.7 ± 53.5 vs. 487.1 ± 88.6 IU, p=0.14) and the duration of ovarian stimulation (8.3 ± 0.6 vs. 7.9 ± 0.5 days, p=0.6) between FPS and LPS (14). That means LPS might represent a logical choice for poor responders.

Currently, there are no evidence for the optimal strategy for LPS in poor responders. According to the multiple waves theory, initiating ovarian stimulation from different stages of luteal phase may be affected by the development of different follicle waves (15). So, an early or delayed LPS initiation may affect ovarian response in patients with ovarian response. Therefore, this study aimed to explore a optimal strategy by evaluating the efficacy of starting gonadotropin on different days during a luteal phase ovarian stimulation. The results of our study may provide clinical care an opportunity to maximize the efficacy and efficiency of LPS for the first time in poor responders.

## Patients and methods

### Study period, population of patients and design

This was a retrospective study on 327 patients conducted between January 2016 and June 2021 at the Reproductive Medicine Center of the First Affiliated Hospital of Sun Yat-sen University. The Institutional Review Board of the clinics approved the study (Approval number [2019]379). All patients with poor ovarian response who consecutively underwent IVF or intracytoplasmic sperm injection (ICSI) treatment in a luteal phase stimulation protocol were candidates to be included. The poor responder was defined according to the Bologna criteria (16): anti-mullerian hormone (AMH) <0.5~1.1 ng/ml, and/or antral follicle count (AFC) <5~7, and/or ≤3 oocytes retrieved from a previous cycle, and/or ≥40 year (at least two out of these conditions should have been satisfied).

### Ovarian stimulation and IVF procedures

All participants were asked to prevent pregnancy by using mechanical contraception or refraining from intercourse during their periovulatory phase. Participants were required to test their urine using an LH kit beginning on cycle day 10. When the LH surge indicator line appeared, they came to the clinic for an ultrasound and/or a serum hormone test. These ultrasound scans, along with serum P concentration testing, helped detect spontaneous ovulation. The ovulation day was set to D0, the next day was D1, and so on. In general, serum P concentration was 0-3ng/ml on D0, 3-6ng/ml on D1, and 6-8ng/ml on D2. For the patients with follicles of <8 mm remaining after ovulation, an (225-300IU) hMG (Anhui Fengyuan Pharmaceutical Co.) IM injection and 2.5-5 mg of letrozole (Jiangsu Hengrui Medicine Co.) were given daily for five consecutive days. Monitoring was performed using transvaginal ultrasound scanning of the ovaries and serum FSH, LH, E_2_, and P measurements. When at least one follicle reached diameters of 18 mm or two follicles reached 17 mm, the final stage of oocyte maturation was induced with 10,000 IU of hCG (Zhuhai Livzon Pharmaceutical Group Inc.). Transvaginal ultrasound–guided oocyte retrieval was performed 36 hours later.

Fertilization was performed by either conventional insemination or ICSI, depending on semen parameters. Embryos were evaluated for fertilization on Day 1 and were morphologically graded on Day 3, Day 5 and Day 6. Cleavage-stage embryos were graded on the third day according to the modified criteria of Khoudje R et al. (17). Blastocysts were evaluated using grading criteria that were previously described by Gardner et al. (18). High-quality cleavage stage embryos (including grade 1 and grade 2 of 7~9 cell blastomere embryos) were vitrified on the third day after oocyte retrieval. All the other embryos were cultured until the blastocyst stage. Blastocysts with good morphology were vitrified on Day 5 or Day 6.

Endometrial preparation and FET were performed in hormone replacement treatment cycles. Initially, 4–6 mg/d estradiol was administered from day 3 of menstrual cycle. When endometrial thickness was ≥ 8 mm, intramuscular injection of progesterone in oil (20 mg per ampoule; Shanghai General Pharmaceutical Factory, Shanghai, China) was started at 40 mg daily for 2 days and increased to 60 mg daily for the following 16 days. Cleavage-stage embryos or blastocysts were thawed on 4 or 6 days of progesterone administration respectively, and embryo transfer was performed on the day of thawing. Serum levels of hCG were measured 14 days or 12 days after embryo transfer respectively, and transvaginal ultrasound was arranged 3-weeks later to confirm a clinical pregnancy. A maximum of two embryos were transferred per cycle.

### Definition of study groups

For each patient in this study cohort, the ovulation day was defined as previously described. Additionally, the ovulation day was set to D0, and the luteal phases were defined as follows: early luteal phase, D0 to D1 post-ovulation; early-mid luteal phase, D2 to D4 post-ovulation; mid-late luteal phase, ≥D5 post-ovulation. Therefore, patients were stratified into three groups according to the gonadotropin initial day in luteal phase: early luteal phase (starting gonadotropin stimulation between D0~D1 post-ovulation), early-mid luteal phase (starting gonadotropin stimulation between D2 to D4 post-ovulation), and mid-late luteal phase (starting gonadotropin stimulation after D5 post-ovulation).

### Outcome measures

The primary outcome of this study was the duration of ovarian stimulation. The median length of ovarian stimulation was 9 days. Thus, we divided patients as the group with shorter ovarian stimulation (≤9 days) and the group with longer ovarian stimulation (≥10 days). Secondary outcomes were the total gonadotropin dose, the number of oocytes retrieved, D3/D5 embryo implantation rate, clinical pregnancy rate, and live birth rate after frozen embryo transfer (FET) cycles. Serum hCG level was checked 12 days or 14 days after embryo transfer, and the level ≥25 IU/L was considered as chemical pregnancy. Clinical pregnancy was diagnosed if a viable intrauterine gestational sac with heart beat was detected by transvaginal ultrasound at 7 weeks’ gestation. Miscarriage was defined as a clinical pregnancy loss before 12 weeks of gestation. In cases of successful pregnancy, progesterone was continued until 10 weeks of gestation. Embryo implantation rate was calculated as the number of embryos with cardiac activity divided by the number of embryos transferred. Ongoing pregnancy was defined as a pregnancy proceeding beyond the 12th gestational week. Live birth was defined as the delivery of a living newborn after the 24th gestational week.

### Statistical analysis

Statistical analyses were carried out using SPSS 25.0 software (SPSS, Inc.). Categorical data are presented as frequency and percentage, and differences were assessed by chi-square test or by Fisher’s exact test for expected frequencies <5. Continuous data were expressed as mean (SD) or median (range) depending on distribution characteristics, and between-group differences were evaluated by the two-sample t-test or Wilcoxon rank-sum test as appropriate. Differences in the means or median of continuous data among three groups were analyzed using one-way ANOVA or Kruskal-Wallis tests, as appropriate. Multivariate logistic regression was performed to analyze the association between day of gonadotropin start and shorter ovarian stimulation (≤9 days). Odds ratios (ORs) and 95% confidence intervals (CIs) were used to measure the strength of associations. Multiple linear regression was performed to analyze the association between the day of initiating gonadotropin and the total Gonadotropin doses. In the regression analysis, the following possible factors were considered as confounders: female age, BMI, AFC, FSH, LH, E2, T, menstrual cycle length, initial Gn dose. Receiver operating characteristic (ROC) analysis was used to evaluate the performance of the prediction models, the area under the curve (AUC) and optimal cutoff levels being determined. A two-tailed value of *P* < 0.05 was considered to denote statistical significance.

## Results

Baseline characteristics of the patients in the three different gonadotropin initial day groups were presented in **Table 1**. There were no differences with regards to female age, duration of infertility, cycle, AFC, AMH, basal endocrine profile, and initial gonadotropin dose in the three groups. BMI was significantly different across the three groups: 22.30 ± 2.86 kg/ml^2^, 21.63 ± 2.69 kg/ml^2^, 21.24 ± 2.75 kg/ml^2^.

**Table 1:** Baseline characteristics and ovarian response in groups with different gonadotropin (Gn) initial phases.

Variables	Early luteal phase (n = 158)	Early-mid luteal phase (n = 110)	Mid-late luteal phase (n = 59)	*P*
Female age (years)	38.91 ± 4.79	38.25 ± 4.61	37.15 ± 4.85	0.051
Infertility duration (years)	4 (2,7)	3 (2,7)	3 (1,7)	0.689
Cycle number	3 (2,5)	3 (2,5)	3 (2,5)	0.691
Infertility type, n (%)				0.116
Primary infertility	42 (26.6)	37 (33.6)	24 (40.7)	
Secondary infertility	116 (73.4)	73 (66.4)	35 (59.3)	
Menstrual cycle length (days)	28.61 ± 6.66	28.35 ± 9.42	27.88 ± 4.22	0.808
Basal FSH level (IU/L)	7.59 (5.86,10.44)	7.91 (5.82,11.12)	7.18 (5.96,10.27)	0.808
Basal LH level (IU/L)	2.70 (2.07,3.64)	2.93 (2.13,3.79)	3.10 (2.37,3.79)	0.170
Basal E_2_ level (pg/mL)	36.50 (25.00,51.25)	35.00 (20.00,48.25)	38.00 (24.00,54.00)	0.659
Basal T level (ng/mL)	0.24 (0.19,0.31)	0.24 (0.19,0.30)	0.26 (0.20,0.35)	0.203
AMH level (ng/mL)	0.64 (0.35,0.98)	0.63 (0.28,1.12)	0.69 (0.33,0.97)	0.989
BMI (kg/m^2^)	22.30 ± 2.86	21.63 ± 2.69	21.24 ± 2.75	0.024*
No. AFC	4 (3,6)	4 (3,7)	5 (3,6)	0.689
Initial Gn dose (IU)	241.33 ± 40.89	240.23 ± 40.79	254.66 ± 44.64	0.070
Duration of Gn stimulation (days)	8 (7,10)	9 (8,10.25)	11 (10,12)	<0.001*
Total dose of Gn (IU)	1993.35 ± 720.31	2282.73 ± 703.38	2764.83 ± 722.26	< 0.001*
E_2_ level on hCG trigger day (pg/mL)	474.50 (238.75,952.25)	401.50 (231.50,782.75)	406.50 (257.75,980.25)	0.717
Number of follicles≥14 mm on hCG trigger day	4 (2,5)	4 (2,6)	3 (2,4.25)	0.331
Number of oocytes retrieved	3 (2,5)	3 (1,5.25)	3 (1.75,4)	0.837
Cycle cancellation rate% (n)	29.7% (47/158)	26.4% (29/110)	27.1% (16/59)	0.761

*Significant difference (P < 0.05).

AFC, antral follicle count; AMH, anti-Müllerian hormone; BMI, body mass index; E_2_, estradiol; FSH, follicle-stimulating hormone; Gn, gonadotropin; hCG, human chorionic gonadotropin; ICSI, intracytoplasmic sperm injection; IVF, in-vitro fertilization; LH, luteinizing hormone; MII, metaphase of meiosis II; T, testosterone.

Continuous variables are presented as mean ± SD or median (quartile 1, quartile 3), categorical variables are presented as frequencies (percentages).

**Table 1** also showed that the duration of gonadotropin stimulation in the early luteal phase group was significantly shorter than those in the early-mid luteal phase group and mid-late luteal phase group [8 (7,10) days, 9 (8,10.25) days, and 11 (10,12) days, respectively; *P*<0.001]. The total gonadotropin dose was significantly less in the early luteal phase group compared with the other groups (1993.35 ± 720.31IU, 2282.73 ± 703.38IU, 2764.83 ± 722.26IU, respectively; *P*<0.001). Despite these differences, E_2_ level on the trigger day and the numbers of follicles with diameter >14 mm on the trigger day were similar across all three groups. Similarly, the numbers of oocytes retrieved were not significantly different across the three groups: 3(2,5) oocytes in the early luteal phase group, 3(1,5.25) oocytes in the early-mid luteal phase group, and 3(1.75,4) oocytes in the mid-late luteal phase group. **Supplemental Table 1** described the oocyte performance in the three groups. Thirty five patients failed to achieve an oocyte, including 15 in the early luteal phase group (9.49%), 12 in the early-mid luteal phase group (10.91%), and 8 in the mid-late luteal phase group (13.56%) (**Supplemental Figure 2**). Number of MII oocytes, number of utilizable embryos, number of high-quality embryos and number of high-quality blastocysts were not significantly different across the three groups.

Next, all cycles were stratified by the median days of stimulation. Accordingly, 184 cycles had a shorter duration of ovarian stimulation (≤9 days), while 143 had a longer duration of ovarian stimulation (≥10 days). **Supplemental Table 2** summarized the baseline characteristics and the ovarian response of the two groups. There were no differences with regards to female age, duration of infertility, cycle, AFC, AMH, basal endocrine profile, and initial gonadotropin dose between the two groups, except for the T level [0.23(0.19,0.30) vs 0.26(0.20,0.33), *P*=0.037]. Gonadotropin initial day was significantly earlier in the shorter ovarian stimulation group compared to the longer stimulation group [1 (1, 2) vs. 3 (1, 5), *P*< 0.001]. Total gonadotropin dose was significantly lower in the shorter ovarian stimulation group compared to the longer stimulation group [1764.67 ± 528.03IU vs 2828.50 ± 590.93IU, *P*<0.001]. The average number of oocytes retrieved in the shorter ovarian stimulation group were slightly lower than that in the longer stimulation group [2(1, 4) vs 3 (2, 5), *P*=0.028]. However, number of MII oocytes, number of utilizable embryos, number of high-quality embryos and number of high-quality blastocysts were not significantly different between the two groups.

A multivariate logistic regression model was used to analyze the association between day of initiating gonadotropin and the shorter duration of ovarian stimulation (≤9 days). Baseline characteristics with statistically significant differences and other variables considered to have an impact on the duration of stimulation were included in the regression model. The variables considered for model selection included female age, BMI, AFC, FSH, LH, E_2_, T, menstrual cycle length, and initial gonadotropin dose. As shown in **Table 2**, the incidences of shorter ovarian stimulation in the early-mid luteal group and the mid-late luteal group were significantly decreased compared with the early luteal group, with the unadjusted odds ratios 0.444 (95% confidence interval [CI]: 0.267-0.739) and 0.113 (95% CI: 0.056-0.228), respectively (*P*-trend<0.001), and the adjusted odds ratios 0.584 (95%CI: 0.327-1.042) and 0.116 (95% CI: 0.049-0.271), respectively (*P*-trend<0.001). The results showed a statistically significant increase in the probability of shorter ovarian stimulation for earlier gonadotropin initiation.

**Table 2:** Association between day of gonadotropin start and the shorter duration of ovarian stimulation (≤9 days) in patients with luteal phase stimulation.

Variables	Unadjusted model		Adjusted model	
	OR (95% CI)	*P* value	OR (95% CI)	*P* value
Gn initial phase				
Early luteal phase (0~1d)	1.00 (ref)		1.00 (ref)	
Early-mid luteal phase (2~4d)	0.444 (0.267, 0.739)	0.02	0.584 (0.327, 1.042)	0.068
Mid-late luteal phase (≥5d)	0.113 (0.056, 0.228)	<0.001	0.116 (0.049, 0.271)	<0.001^*^
Per-day increase	0.650 (0.569, 0.741)	<0.001	0.670 (0.575, 0.782)	<0.001^*^

*Significant difference (P < 0.05).

CI, confidence interval.

The variables in the adjusted model were adjusted for potential confounders: female age, BMI, AFC, FSH, LH, E_2_, T, menstrual cycle length, initial Gn dose.

**Table 3** presented the results of unadjusted and adjusted analyses for the association between day of gonadotropin start and total gonadotropin dosage in patients conducting luteal phase stimulation. Multiple linear regression were conducted of patient baseline and treatment characteristics associated with the total gonadotropin dose, of each variables hypothesized or proven to be associated with the total gonadotropin dose. These characteristics included female age, BMI, AFC, FSH, LH, E2, T, menstrual cycle length, and initial gonadotropin dosage. After controlling for confounders, our results showed that the total gonadotropin dosages among the early-mid luteal group and the mid-late luteal group were significantly increased compared to the early luteal group, with the unadjusted β value 289.373 (95% CI: 114.698, 464.047) and 771.476 (95% CI: 556.860, 986.092), respectively, and a 147.949 IU(95%CI: 110.344, 185.554) per-day increase (*P <*0.001). After adjusting confounding factors, the adjusted β value were 228.563 (95%CI: 44.885, 412.240) and 563.658 (95% CI: 326.473, 800.843), respectively, with an 113.200 IU (95%CI: 70.469, 155.930) per-day increase (*P <*0.001). The results showed a statistically significant increase in the total gonadotropin dosage for delayed gonadotropin initiation.

**Table 3:** Association between day of gonadotropin start and total gonadotropin dosage in patients with luteal phase stimulation.

Variables	Unadjusted model		Adjusted model	
	β (95% CI)	*P* value	β (95% CI)	*P* value
Gn initial phase				
Early luteal phase (0~1d)	0.00 (ref)	–	0.00 (ref)	–
Early-mid luteal phase (2~4d)	289.373 (114.698, 464.047)	0.01	228.563 (44.885, 412.240)	0.015^*^
Mid-late luteal phase (≥5d)	771.476 (556.860, 986.092)	<0.001	563.658 (326.473, 800.843)	<0.001^*^
Per-day increase	147.949 (110.344, 185.554)	<0.001	113.200 (70.469, 155.930)	<0.001^*^

*Significant difference (P < 0.05).

CI, confidence interval.

The variables in the adjusted model were adjusted for potential confounders: female age, BMI, AFC, FSH, LH, E_2_, T, menstrual cycle length, initial Gn dose.

ROC curves were constructed to assess the strength of day of gonadotropin start for predicting the duration of ovarian stimulation in LPS. The result showed an AUC of 0.712 (0.655-0.769) with a sensitivity of 0.601, a specificity of 0.783, suggesting a good predictive performance. As illustrated in **Supplemental Table 3** and **Supplemental Figure 1**, the optimum cut-off value of gonadotropin initial day for prediction of shorter ovarian stimulation was 2.5, which indicates that cycles with gonadotropin initiation during 2 days post-ovulation may be more likely to present a shorter ovarian stimulation.

Totally, 152 patients underwent FET in 184 cycles (**Supplemental Figure 2**). The pregnancy outcomes were displayed in **Table 4**. The average number of embryos transferred was comparable among the different gonadotropin initial day groups (1.56 ± 0.52, 1.60 ± 0.52 and 1.54 ± 0.50, *P*=0.794). There were no significant differences in clinical pregnancy rate among three groups: 25.9% in the early luteal phase group, 26.2% in the early-mid luteal phase group, and 23.8% in the mid-late luteal phase group (*P*=0.957). The D3 and D5 embryo implantation rates showed no significant differences among these groups: 17.0% and 46.7% in the early luteal phase group, 15.9% and 40.0% in the early-mid luteal phase group, and 20.0% and 22.2% in the mid-late luteal phase group. There were no significant differences in the miscarriage rate and live birth rate among the three groups. It seemed to be a tendency for the embryos obtained in the early luteal phase group had a slightly higher blastocyst implantation rate, but the difference did not reach statistical significance.

**Table 4:** Clinical outcomes of frozen-thawed embryo transfers originating from different gonadotropin initial phase groups.

Outcome		Early luteal phase	Early-mid luteal phase	Mid-late luteal phase	*P value*
FET cycle n		81	61	42	
Embryo transferred per cycle		1.56±0.52	1.60±0.52	1.54±0.50	0.794
Implantation rate per embryo transferred % (n)	D3 embryo transfer	17.0 (19/112)	15.9 (14/88)	20.0 (11/55)	0.815
	D5 embryo transfer	46.7 (7/15)	40.0 (4/10)	22.2 (2/9)	0.599
Clinical pregnancy rate per transfer % (n)		25.9 (21/81)	26.2 (16/61)	23.8 (10/42)	0.957
Miscarriage rate % (n)		19.0 (4/21)	12.5 (2/16)	10.0 (1/10)	0.759
Ongoing pregnancy rate per transfer n (%)		21.0 (17/81)	23.0 (14/61)	21.4 (9/42)	0.960
Live birth rate per transfer n(%)		14.8 (12/81)	18.0 (11/61)	19.0 (8/42)	0.800

## Discussion

In the study, we found that the day of gonadotropin initiation in women with predicted poor ovarian response undergoing a LPS affected the efficiency of COS. An earlier gonadotropin initiation was associated with an increased probability of shorter duration of stimulation, while delayed gonadotropin initiation showed an 113.200 IU increase (95%CI: 70.469, 155.930) per-day in the total gonadotropin dosage. Meanwhile, there is no significant difference among the pregnancy outcomes of the three groups with different gonadotropin initiation time.

To date, few studies have evaluated the ovarian responsiveness to gonadotropin in LPS protocol according to the timing for starting COS. A retrospective cohort study in cancer patients showed that the length of the cycle and daily gonadotropin dosage was not affected by the timing of starting COS, whether COS was started in the early or mid-luteal phase (19). While from the results of other studies, it seems that the later the stimulation started, the longer the stimulation periods were, which is consistent with the conclusion of this study. In Martinez’s study, the stimulation started on the D15 of menstrual cycle, with 9.89 ± 1.27 days spent (7). In Wang’s study, the stimulation started 1 ~ 3 days after ovulation and lasted 10.4 ± 1.8 days (6). In the study of Buendgen et al, the stimulation started in the D19~21 of a menstrual cycle, lasting 11.7 ± 1.57 days (5).

Our conclusions can be explained by follicular wave theory, providing clinical evidence for follicular development dynamics in luteal phase. Follicular development occurs in a multiple wave-like pattern in both domesticated animal species and human. The evidence that multiple follicular waves exist during an ovarian cycle opened a new way for the treatment of patients with poor prognosis. Baerwald et al. (15) showed that there were two or three waves of follicle recruitment during one menstrual cycle, and the ovulatory wave appeared in the early follicular phase and anovulatory waves developed in the luteal phase, with peaks in follicle number were often detected on the day of ovulation. Moreover, previous study also showed that the physiologic changes in the follicles in luteal phases, with lower numbers of healthy follicles per ovary were found in the midluteal phase than in the early luteal phase [0.3 (0-2) vs. 1.6 (0-4)], and less testosterone relative to androstenedione during the midluteal phase compared to the early luteal phase (20). Accordingly, granulosa cells from the early luteal phase follicles may be more responsive to FSH than those in cells from midluteal phase follicles, resulting in a better response to ovarian stimulation. In another word, it may imply that follicle sensitivity to the Gonadotropin stimulation may be reduced during the mid-luteal phase.

Based on the above knowledge on folliculogenesis, we hypothesized that initiating ovarian stimulation at the time of emerging or peak of antral follicle wave may be more effective. Moreover, the action of androgen should be considered during LPS. The health of the small antral follicles is driven primarily by androgens, which contributes to granulosa cell mitosis, sensitivity to FSH, and resistance to atresia. In contrast, elevated androgens in the late antral to pre-ovulatory follicle have a negative impact on follicle health. FSH and estrogen’s actions on granulosa cells become important for extending follicle growth past the early antral follicle transition point. Androgen production induced by LH surge during periovulatory stage may act on folliculogenesis and increase the sensitivity to FSH. It was shown that androgens induce the expression of FSH receptor in granulosa cells to potentiate the effect of FSH and exert paracrine regulation on follicular maturation, on the other hand reduce follicular atresia (21, 22). Letrozole is a targeted non-steroidal aromatase inhibitor, which is a potentially important factor in LPS. It blocks aromatization of androgens into oestrogens and releases the hypothalamic–pituitary axis from negative estrogen feedback, increasing the secretion of FSH by the pituitary gland, whereas the increase of intraovarian androgens enhances early follicular growth and results in improved ovarian responsiveness (23, 24). It is worth noting that Kuang et al. considered it necessary to add letrozole when stimulating in luteal phase to increase the sensitivity of follicles to gonadotropins (4, 25). Thus, in the present study of the cohort patients with poor-prognosis met Bologna criteria, we added letrozole for five days since the gonadotropin initiation day, the same protocol as the Kuang et al. We defined shorter ovarian stimulation (≤9 days) and longer ovarian stimulation (≥10 days) according to the cohorts’ median number of days for ovarian stimulation. We provided evidence that early initiation of gonadotropin administration after ovulation resulted in a shorter duration of ovarian stimulation, fewer total gonadotropin doses, with comparable oocytes retrieved and comparable embryos obtained. In addition, early initiation in LPS may allow us to avoid oocyte retrieval in the next menstruation.

We postulated that day of gonadotropin initiation might be an indicator to COS to guide clinical treatment. To evaluate this possibility, the independent predictive value of gonadotropin initial day was determined by the receiver operating characteristic curve analysis. Patients starting gonadotropin stimulation within 2 days post-ovulation (gonadotropin initial day < 2.5) were more likely to have a shorter ovarian stimulation.

In the present study, no differences were observed in the subsequent evolution of the oocytes obtained between the three initial groups. Although there was a trend for the embryos obtained in the early or early-mid luteal phase group to have a higher blastocyst implantation rate than the mid-late luteal phase group, no impact of the day of gonadotropin start on oocyte competence was found in our study. The clinical pregnancy rates, implantation rates, miscarriage rates, live birth rates were similar among the three groups. However, there were 83 patients who asked for embryo accumulation did not undergo FET procedure yet, hence, the complete clinical results could not be obtained. Due to the relatively small sample size, further research and follow-up are required.

To the best of our knowledge, this was the first study comparing the efficacy and efficiency of LPS with different gonadotropin start days in a population of poor responders. Our study had several strengths. First, most studies on the luteal phase stimulation have been based on data compared with follicular phase stimulation. However, this study focused on LPS efficacy with different gonadotropin initial days as a new direction in this field, optimizing the application of LPS protocol among poor responders. Second, our results provided new evidence for the follicular dynamics in luteal phase, contributing to the development of the mechanism theories. Moreover, the implication of our study was the possibility that the early gonadotropin initiation after ovulation could improve the efficiency and convenience levels of LPS cycles in patients with poor responder.

Several limitations of our study should be considered. First, the retrospective study might be affected by selection bias that which patients at our center were recommended to LPS protocol. Therefore, it is unknown whether similar results would be observed in patients of normal responder or high responder. Second, due to the retrospective nature of the study, patients could not be randomized to a specific treatment group. Third, failure to know the treatment outcome of non-transferred frozen embryos due to exceeding the follow-up time may potentially bias the study results. Lastly, our results were based on a single-center, and a relatively small sample size population. Thus, caution should be taken in generalizing this finding and larger multi-center randomized studies are mandatory to confirm the best option for gonadotropin initial day of LPS in this population. More studies need to be conducted in the future to confirm the safety of LPS, in terms of ovarian (and follicular) environment as well as clinical, peri-natal and post-natal outcomes.

To conclude, our study indicated that an earlier gonadotropin initiation after ovulation declined the duration and consumption of gonadotropin in ovarian stimulation, improving the efficacy of LPS cycles in poor responders.

## Data availability statement

The original contributions presented in the study are included in the article/**Supplementary Material**. Further inquiries can be directed to the corresponding author.

## Ethics statement

The studies involving human participants were reviewed and approved by the First Affiliated Hospital of Sun Yat-sen University (Approval number[2019]379). The patients/participants provided their written informed consent to participate in this study.

## Author contributions

JH contributed in the study design, data analysis, and manuscript writing. LL contributed in the data acquisition and manuscript writing. YW contributed in the data acquisition and data analysis. BM and YL contributed in the data acquisition. CZ contributed in the manuscript editing. YX contributed in conception of the idea and study design, and is responsible for the integrity of the work. All authors contributed to the article and approved the submitted version.

## Funding

This work was funded by the National Natural Science Foundation of China (82071716) and the Guangdong Basic and Applied Basic research (2022A1515012599).

## Acknowledgments

The authors thank Dr. AiPing Fang for assistance with statistical analysis and Dr. James Segars for manuscript modifying.

## Conflict of interest

The authors declare that the research was conducted in the absence of any commercial or financial relationships that could be construed as a potential conflict of interest.

## Publisher’s note

All claims expressed in this article are solely those of the authors and do not necessarily represent those of their affiliated organizations, or those of the publisher, the editors and the reviewers. Any product that may be evaluated in this article, or claim that may be made by its manufacturer, is not guaranteed or endorsed by the publisher.

## Supplementary material

Supplementary Figure 1 AUC, area under curve; CI, confidence interval. Area under curve of ROC analysis for day of gonadotropin start and specific cut-off values for prediction of duration of stimulation in LPS

Supplementary Figure 2 Flowchart of the study. Enrollment and analysis of patients.
