# Supplementary material for: The earlier the initiation of gonadotropin in poor responders in luteal phase stimulation protocols, the better
Source: Front Endocrinol (Lausanne). 2022 Nov 16;13:979934. doi: 10.3389/fendo.2022.979934 (PMC9709114; doi:10.3389/fendo.2022.979934)
Supplement: Supplementary Figure 1 — AUC, area under curve; CI, confidence interval. Area under curve of ROC analysis for day of gonadotropin start and specific cut-off values for prediction of duration of stimulation in LPS [file DataSheet_1.docx]

**Supplemental Figure 1.**


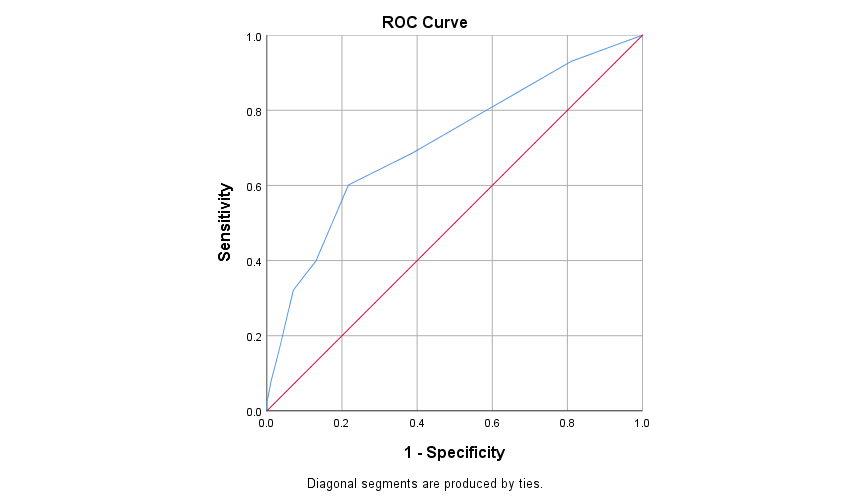


**Supplemental Figure 2.** Flowchart of the study.

Patients conducted IVF/ICSI with LPS protocol

(n=411)

Not meeting inclusion criteria (n=84)

Patients with poor prognosis enrolled

(n=327)

Patients completing ovarian stimulation during luteal phase

(n=327)

No oocyte retrieved

(n=35)

Patients with ≥1 oocyte retieved

(n=292)

No utilizable embryos for cryopreservation

(n=57)

Patients with embryos for FETs

(n=235)

Patients undergoing FET cycles (n=152)

(184 FET cycles)

Did not perform FETs to date

(n=83)

Follow-up pregnancy outcomes for FET cycles

(184 FET cycles )

( FET cycles)
