# Supplementary material for: The earlier the initiation of gonadotropin in poor responders in luteal phase stimulation protocols, the better
Source: Front Endocrinol (Lausanne). 2022 Nov 16;13:979934. doi: 10.3389/fendo.2022.979934 (PMC9709114; doi:10.3389/fendo.2022.979934)
Supplement: Supplementary Figure 2 — Flowchart of the study. Enrollment and analysis of patients. [file Table_1.docx]

**Supplemental Table 1.** Embryological data in patients with different gonadotropin initial day.

| Variables | Early luteal phase  (n =143) | Early-mid luteal phase  (n =98) | Mid-late luteal phase  (n =51) | *P* ***value*** |
| --- | --- | --- | --- | --- |
| Number of oocytes retrieved | 3(2,5) | 3(2,6) | 3(2,4) | 0.548 |
| Number of MII oocytes | 2(1,4) | 3(1,4) | 2(1,4) | 0.523 |
| Fertilization rate (%) | 100(66.67,100) | 100(69.05,100) | 100(80,100) | 0.366 |
| Number of utilizable embryos | 1(1,2) | 2(1,2) | 1(1,2) | 0.240 |
| Number of high-quality embryos | 1(0,1) | 1(0,2) | 1(0,1) | 0.257 |
| Number of high-quality blastocysts | 0(0,0) | 0(0,0) | 0(0,0) | 0.850 |

*Significant difference (P < 0.05)

Continuous variables are presented as the median (quartile 1, quartile 3).

Note: Only patients with oocytes available were included.

**Supplemental Table 2.** Baseline characteristics and embryological data according to different duration of ovarian stimulation in patients conducting luteal phase stimulation (LPS)

|  | Duration of ovarian stimulation (days) | |  |
| --- | --- | --- | --- |
|  | ≤9 (n=184) shorter | ≥10 (n=143) longer | *P* value |
| Female age (years) | 38.59±4.76 | 38.09±4.79 | 0.346 |
| Infertility duration (years) | 3(2,7) | 3(2,7) | 0.412 |
| Cycles number | 3(2,5) | 3(2,5) | 0.800 |
| Menstrual cycle length (days) | 28.41±6.45 | 28.37±8.42 | 0.955 |
| BMI (kg/m2) | 21.93±2.70 | 21.82±2.95 | 0.717 |
| No. AFC | 4(3,6) | 5(3,7) | 0.675 |
| AMH level (ng/mL) | 0.64(0.31,0.98) | 0.64(0.35,0.99) | 0.512 |
| Basal FSH level (IU/L) | 7.63(5.57,10.74) | 7.73(6.07,10.27) | 0.699 |
| Basal LH level (IU/L) | 2.85(2.17,3.59) | 2.90(2.08,3.88) | 0.761 |
| Basal E2 level (pg/mL) | 38.50(24.00,55.50) | 34.00(24.00,46.00) | 0.207 |
| Basal T level (ng/mL) | 0.23(0.19,0.30) | 0.26(0.20,0.33) | 0.037* |
| Gn initial day(days) | 1(1,2) | 3(1,5) | <0.001* |
| Early luteal, n (%) | 113 (61.4) | 45 (31.5) | <0.001* |
| Early-mid luteal, n (%) | 58(31.5) | 52 (36.4) |  |
| Mid-late luteal, n (%) | 13(7.1) | 46 (32.2) |  |
| Initial Gn dose (IU) | 242.83±38.23 | 244.06±46.06 | 0.792 |
| Total dose of Gn (IU) | 1764.67±528.03 | 2828.50±590.93 | <0.001* |
| E_2_ level on hCG trigger day (pg/mL) | 404.00(229.00,950.00) | 488.00(265.00,955.00) | 0.314 |
| Number of follicles measuring≥14 mm on hCG trigger day | 4(2,5) | 3(2,6) | 0.841 |
| Number of retrieved oocytes | 2(1,4) | 3(2,5) | 0.028* |
| Number of MII oocytes | 2(1,4) | 2(1,4) | 0.255 |
| Fertilization rate (%) | 100(66.67,100.00) | 100(75.00,100.00) | 0.784 |
| Number of utilizable embryos | 1(0,2) | 1(1,2) | 0.220 |
| Number of high-quality embryos | 1(0,1) | 1(0,1) | 0.587 |
| Number of high-quality blastocysts | 0(0, 0) | 0 (0, 0) | 0.939 |

* Significant difference (P < 0.05)

AFC, antral follicle count; AMH, anti-Müllerian hormone; BMI, body mass index; E2, estradiol; FSH, follicle stimulating hormone; Gn, gonadotropin; HCG, human chorionic gonadotropin; ICSI, intracytoplasmic sperm injection; IVF, in-vitro fertilization; LH, luteinizing hormone; MII, metaphase of meiosis II; T, testosterone.

Continuous variables are presented as as mean±SD or median (quartile 1, quartile 3), categorical variables are presented as frequencies (percentages).

**Supplemental Table 3.** Area under curve of ROC analysis for gonadotropin initial phase and specific cut-off values for the prediction of duration of ovarian stimulation in LPS

| **Cut-off value** | **AUC** | **95%CI** | **p-value** | **Sensitivity** | **Specificity** |
| --- | --- | --- | --- | --- | --- |
| 2.5 | 0.712 | (0.655-0.769） | <0.001* | 0.601 | 0.783 |

* Significant difference (P < 0.05)

AUC, area under curve; CI, confidence interval.
